# Fractional curative killing of pyronaridine or artesunate combinations with tafenoquine, 4-aminoquinolines, or azithromycin in a murine malaria-luciferase model

**DOI:** 10.1128/aac.00247-25

**Published:** 2025-07-31

**Authors:** Gayoung Lee, Janessa Sochima Aneke, David J. Sullivan

**Affiliations:** 1 W. Harry Feinstone Department of Molecular Microbiology and Immunology, Johns Hopkins Bloomberg School of Public Health 25802, Baltimore, Maryland, USA; The Children's Hospital of Philadelphia, Philadelphia, Pennsylvania, USA

**Keywords:** *Plasmodium*, malaria, pyronaridine, artemisinin tafenoquine, combination chemotherapy, chloroquine, amodiaquine, azithromycin

## Abstract

Malaria drug interactions in cytostatic or inhibitory *in vitro* assays or suppression models *in vivo* can be different than curative killing interactions. In the pharmacodynamic high parasitemia *Plasmodium berghei* ANKA-luciferase mouse blood-stage model, we investigated curative interaction analysis of multiple, daily dosed, short half-life, artesunate or single-dose, long half-life, pyronaridine against three single-dose, long half-life, quinolines—chloroquine, amodiaquine, and tafenoquine. Positive or negative parasiticidal activity measured by parasite reduction rate in the days post-treatment correlated nonspecifically to final curative interactions. Tafenoquine/artesunate and pyronaridine/amodiaquine also had fractional combination curative doses of 0.83 and 0.93, with the rest of the interactions closer to neutral at 0.9–1.1. All tested combinations are in the additive drug interaction range. Time to return of initial parasitemia in subcurative regimens was also imprecise for the prediction of cure with combinations. Short blood half-life azithromycin, requiring multiple daily doses, was additive to artesunate or pyronaridine in fractional curative dose combination killing. Murine malaria high parasitemia drug interactions at the curative metric *in vivo* are a potential benchmark for human studies.

## INTRODUCTION

Drug combinations are important to lower doses of drug and combat drug resistance by targeting diverse pathways (1, 2). While quinine was used effectively for centuries with sporadic and unsustained *Plasmodium falciparum* resistance, other quinolines used as single drugs developed resistance (3). Malaria drugs are characterized by different rates of kill or parasite reduction ratios in humans, with the artemisinin demonstrating an approximately 4 log drop in parasitemia, the 4-aminoquinolines a 3 log drop, quinine and pyrimethamine, and sulfadoxine a 2 log drop, and the antibiotics have been labeled with a single log drop in parasitemia over a 48-hour period (4, 5). In human clinical studies, clindamycin, the macrolides, and doxycycline have a generational delay in decreasing parasites, but later still have faster log drop approaching 2–3 logs (6).

*P. falciparum* drug interactions studies at the bench measure the 50% suppression inhibition concentration compared with untreated drug control, which does not measure parasite reduction or killing operable in the human host (5, 7, 8). Tafenoquine was found to be antagonistic to artemisinins in the dormant *Plasmodium vivax* human clinical trial (9). There is a growing consensus that drug action alone or in combination on inhibition is different from action alone or in combination on killing (5, 8, 10). Again, most murine animal models of drug combination have used the 4- to 7-day suppression test dosing drug within 1–2 days after infection compared with untreated control, which increases 4 logs over 4 days (11, 12). Rare combination studies use a low parasite inoculum to follow both suppression and cure (13). Drugs that suppress parasite multiplication rate to no multiplication or a tenfold increase over 4 days without actually lowering parasitemia from a 200,000 infected erythrocyte inoculum will produce a 99.99–99.9% lower parasite level compared with untreated controls that reach near a million per μL in the 2 mL mouse blood volume. The humanized mouse model allows pharmacodynamics of *P. falciparum* in mice, but limitations are less than robust growth and altered pharmacokinetics (10).

Here, we sought to investigate drug interactions based on curative killing, starting at a high parasitemia of near a million parasites per μL, which approximates 2 billion total parasites in 2 mL blood volume of the mouse. This work compares artesunate or pyronaridine with the 4-aminoquinolines, chloroquine, amodiaquine, and pyronaridine, the newly approved 8-aminoquinolines that had blood-stage activity tafenoquine and the macrolide azithromycin. Artesunate and azithromycin have short half-lives in the blood and were dosed daily at human equivalent doses of 50 mg/kg in the mouse equal to a daily human total dose of 250 mg. The subcurative doses were in number of days rather than traditional mg/kg. The quinolines were able to be dosed in a single dose in ranges to achieve curative killing of the high number of parasites.

## RESULTS

### Minimum curative dose

**Fig 1:**
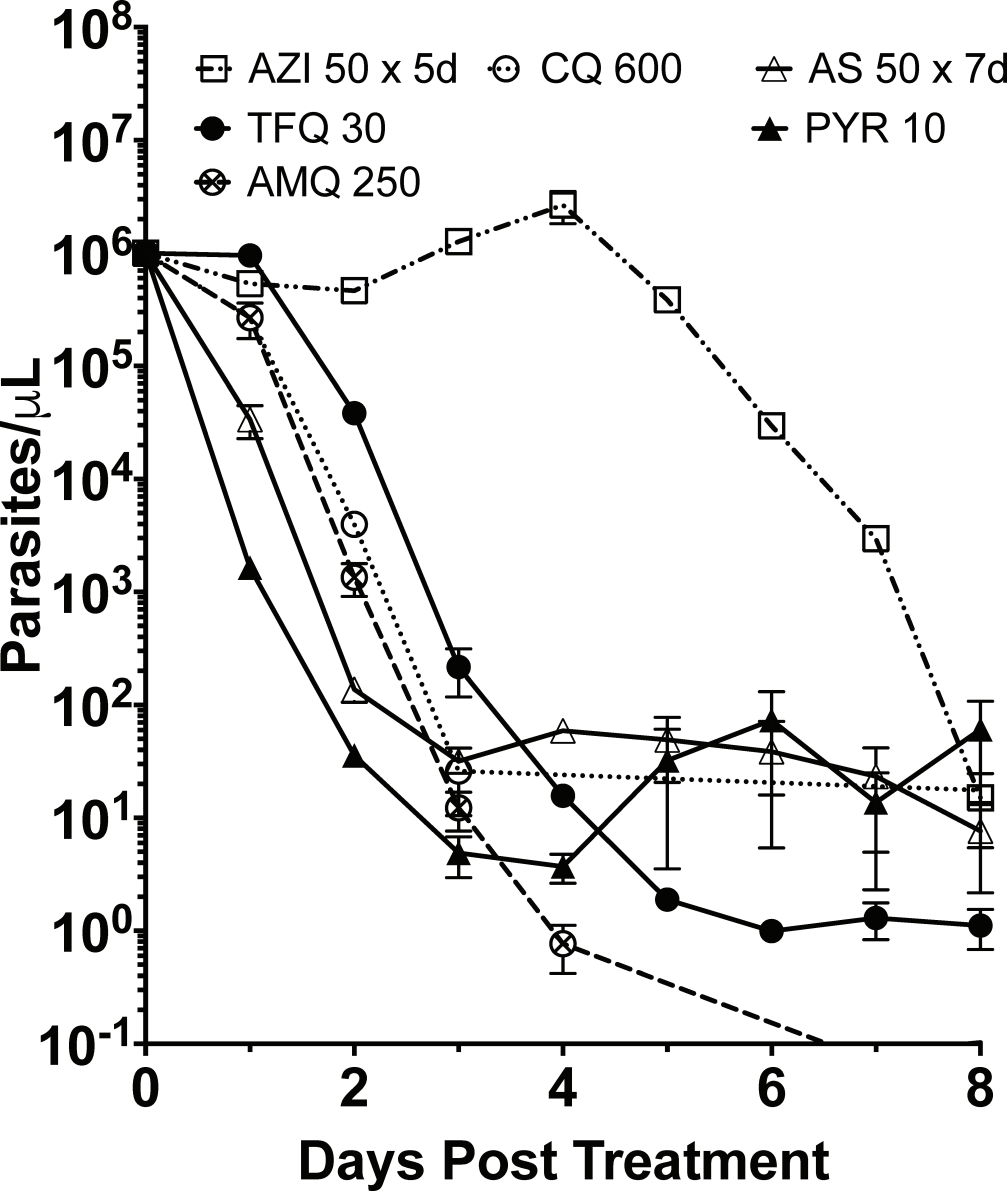
Pharmacodynamics of minimal curative drug dose. Artesunate and pyronaridine demonstrate rapid parasite clearance before 24 hours, with azithromycin having a 4-day delay in significant parasite clearance. Single-dose oral doses of tafenoquine 30 mg/kg (TFQ 30), amodiaquine 250 mg/kg (AMQ 30), and chloroquine 600 mg/kg (CQ 600) were dosed 4–5 days after blood inoculation. Single-dose pyronaridine 10 mg/kg (PYR 10) and daily dosed artesunate 50 mg/kg (AS 50 × 7 days) for 7 days were given by intraperitoneal administration. Oral azithromycin 50 mg/kg (AZI 50 × 5 days) was dosed daily for 5 days. Female Balb/cj mice (*n* = 3) were inoculated with approximately 1 million infected erythrocytes from a donor mouse 4–5 days before drug dosing at about 1 million parasites per μL. Data are represented as mean ± SEM.

In the high parasitemia murine malaria model, the long half-life quinolines are able to cure the initial high parasitemia near one million per μL (a billion per mL) with increasing single doses of drugs. The curative dose ranged from a single dose of 10 mg/kg in the case of pyronaridine tetraphosphate, 30 mg/kg tafenoquine succinate, 250 mg/kg amodiaquine dihydrochloride, and 600 mg/kg chloroquine diphosphate (Fig. 1). We dosed on salt weight, with curative free base equivalents of 5.6, 24, 190, and 372 mg/kg respectively. For the rest of the paper, dosing will be given in total salt in mg/kg. For artesunate and azithromycin that have shorter half-lives in the blood, we dosed by increasing duration in days, with a 7-day duration of 50 mg/kg artesunate being curative (no recrudescent parasites after 30 days) and 5 days of azithromycin curative (2 days shorter than artesunate).

The minimal curative dose of pyronaridine cleared more parasites, expressed in a parasite reduction ratio of 3 log_10_ cleared in 24 and 5 log_10_ in 72 hours post-treatment initiation (Table 1). Artesunate was the next fastest at reduction in the initial 24-hour window of 1.4 log_10_ and 4.4 in 72 hours. Chloroquine and amodiaquine had a slower 24-hour reduction of 0.6, but then at 72 hours, they achieved a 4.5 to 4.1 log_10_ reduction, respectively. Tafenoquine did not appreciably reduce parasites in the first 24 hours, but then mirrored the rate of chloroquine with a 24-hour lag time. Azithromycin did not show a significant measurable decrease until after day 5, where the rate was then relatively fast, achieving cure in 5 days, in contrast to 7 days for artesunate.

**TABLE 1:** Parasite reduction ratio on log_10_ scale by day after treatment initiated

Day post Tx	ART PRR Log_10_	PYR PRR Log_10_	TAF PRR Log_10_	AMQ PRR Log_10_	CQ PRR Log_10_	
1	1.4	3.0	0.1	0.6	0.6	
2	3.4	3.8	1.0	2.1	2.7	
3	4.4	5.0	2.8	4.1	4.5	

Day post Tx	ART PYR PRR Log_10_	ART-TAF PRR Log_10_	ART-AMQ PRR Log_10_	ART-CQ PRR Log_10_	AMQ-CQ PRR Log_10_	
1	2.1	3.0	2.1	1.0	0.5	
2	4.6	4.7	4.3	3.9	2.4	
3	6.0	4.8	5.2	5.3	4.6	

Day post Tx	PYR-TAF PRR Log_10_	PYR-AMQ PRR Log_10_	PYR-CQ PRR Log_10_	AZI PRR Log_10_	AZI-ART PRR Log_10_	AZI-PYR PRR Log_10_
1	1.5	3.2	0.4	0.2	1.2	2.8
2	2.7	6.1	2.9	0.4	3.1	4.0
3	4.3	5.5	5.1	0.0	3.8	6.1
4	–^*a*^	–	–	−0.2	3.3	6.9
5	–	–	–	0.6	3.2	6.8
6	–	–	–	1.8	3.7	6.9
7	–	–	–	2.6	3.7	6.8
8	–	–	–	4.4	3.9	6.7
9	–	–	–	–	6.1	6.8

^*a*^ “–” indicates that the parasites fall to undetectable range.

### Curative combination interactions

**FIG 2:**
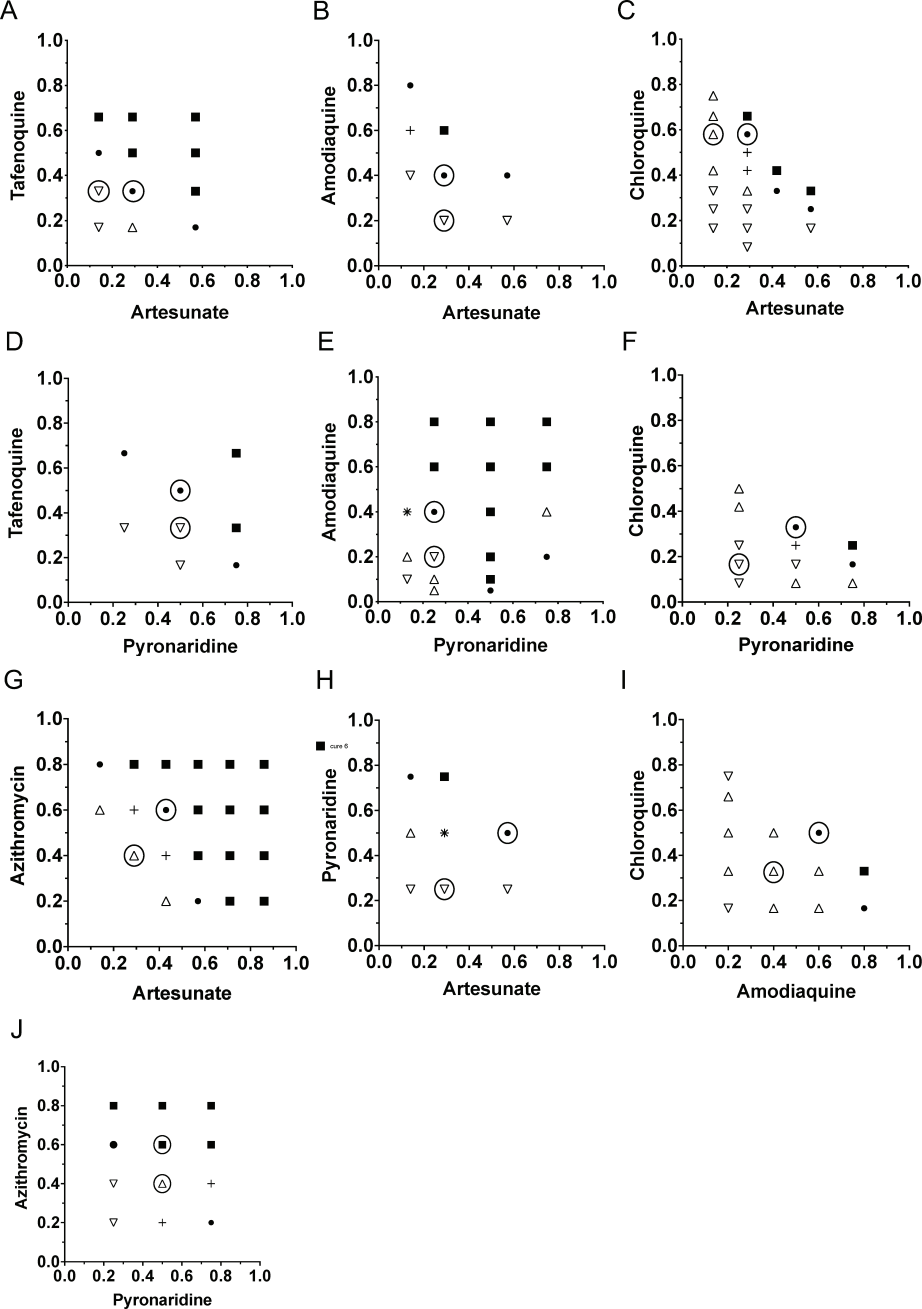
Checkerboard combination drugs to scale of fractional curative single drugs. Combination pairs are tafenoquine/artesunate (*n* = 45 mice) (**A**), amodiaquine/artesunate (*n* = 24 mice) (**B**), chloroquine/artesunate (*n* = 60 mice) (**C**), tafenoquine/pyronaridine (*n* = 24 mice) (**D**), amodiaquine/pyronaridine (*n* = 57 mice) (**E**), chloroquine/pyronaridine (*n* = 36 mice) (**F**), azithromycin/artesunate (*n* = 63 mice) (**G**), pyronaridine/artesunate (*n* = 24 mice) (**H**), chloroquine/amodiaquine (*n* = 39 mice) (**I**), and azithromycin/pyronaridine (*n* = 36 mice) (**J**). The *x*- and *y*-axes represent the fraction of the curative dose of drugs alone. Filled circles and squares represent cure at 30 days for all three mice. + is 2/3 mice cure, * is 1/3 mouse cure, triangle is return to initial parasitemia in under 12 days and inverted triangle is return to initial parasitemia 12 days or over 12 days for all three mice. The empty large circles around the filled symbols or empty triangles represent the curative or noncurative combinations selected for Fig. 4 and 5.

**FIG 3:**
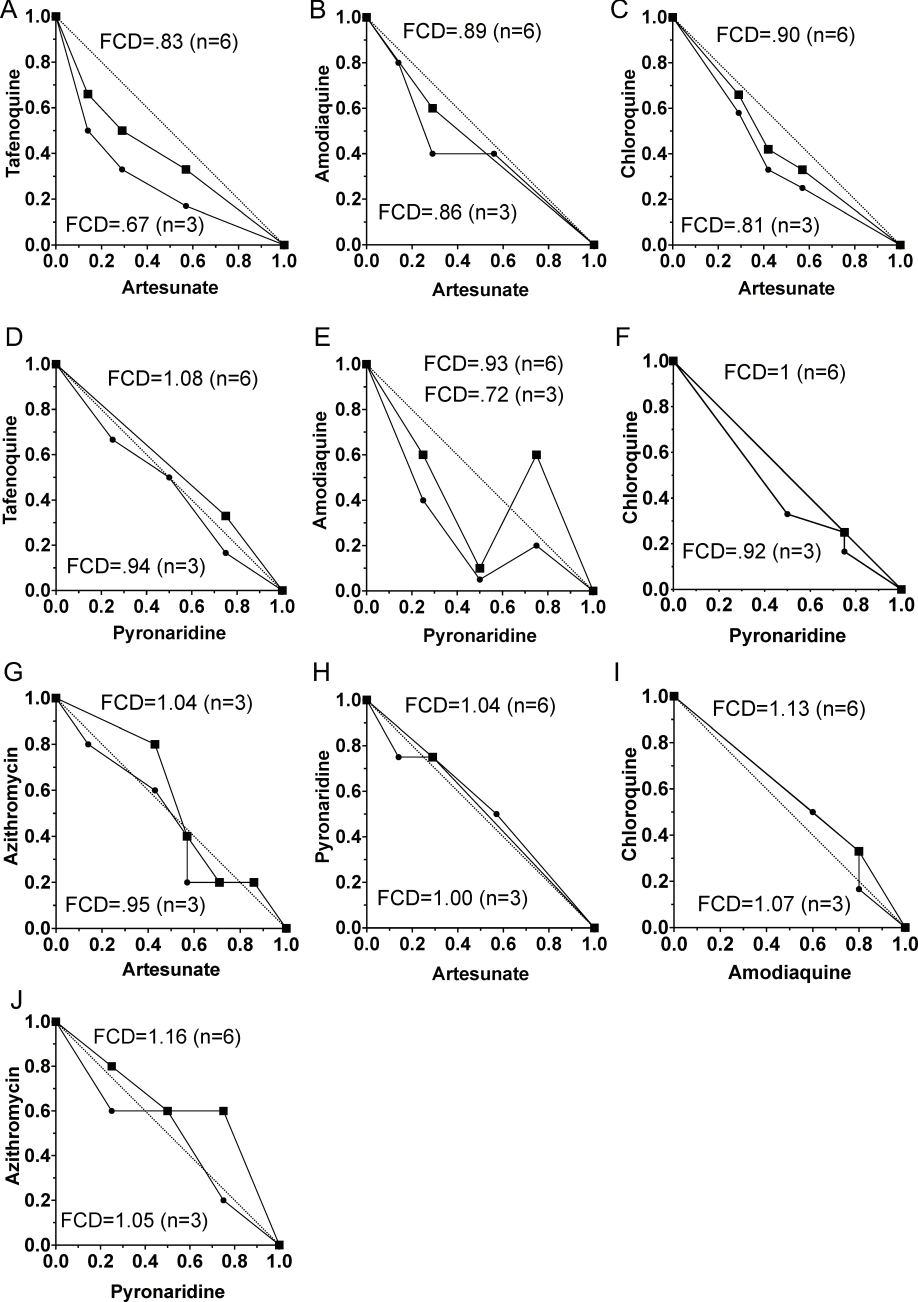
Isobolograms of the fractional curative doses. All tested combinations are in the additive drug interaction range. Combination pairs are tafenoquine/artesunate (**A**), amodiaquine/artesunate (**B**), chloroquine/artesunate (**C**), tafenoquine/pyronaridine (**D**), amodiaquine/pyronaridine (**E**), chloroquine/pyronaridine (**F**), azithromycin/artesunate (**G**), pyronaridine/artesunate (**H**), chloroquine/amodiaquine (**I**), and azithromycin/pyronaridine (**J**). The small filled circles represent combinations with three mice and the large black squares represent higher *y*-axis drug concentration, combining two curative groups graphically at higher concentration with six mice in a group. The line of identity is shown with a dotted line. Synergy would be a fractional curative dose less than 0.5, which was not achieved with any of the combinations. Additive is in the range of 0.51–1.99.

Mice were dosed with subcurative doses in combination in a checkerboard pattern adopted from Chou and Talalay Fractional Inhibition Combination Index (14, 15) for Fractional Curative Doses. Ten dual drug combination interactions were tested with 8–21 (average 14) combinations with 9–40 mice cured (average 24), and 9–38 not cured (average 17) (Table 2). The sum of fractional curative doses for each drug in the combinations was averaged to obtain the fractional curative index (Table 3). The initial design of three mice in a group was computationally altered for six mice in a curative group at the next highest quinoline concentration or number of days dosed for azithromycin or artesunate (Table 3). All the combinations were in the additive range. Despite a 24-hour delay in tafenoquine parasite reduction, tafenoquine and artesunate measured a lower fractional curative dose of 0.83 of all the artesunate pairs tested (Fig. 2 and 3). These checkerboard pairs were designed with sub-curative doses of drugs with the days to return to initial parasitemia of 1 million per μL in the lower left portions of the grid, along with curative doses in the upper right. These values are slightly underestimated with the fractional doses tested for artesunate limited to 24-hour fractions (14%) and 5 mg incremental tafenoquine doses (16%). Artesunate in this model was additive for fractional curative dose above 0.8 for amodiaquine, chloroquine, pyronaridine, and azithromycin.

**TABLE 2:** Combinations tested for cure or no cure

Combination	Dose combinations	Total mice	# cured	# no cure
Tafenoquine/artesunate	15	45	36	9
Amodiaquine/artesunate	8	24	14	10
Chloroquine/artesunate	20	60	22	38
Tafenoquine/pyronaridine	8	24	15	9
Amodiaquine/pyronaridine	19	57	40	17
Chloroquine/pyronaridine	12	36	11	25
Azithromycin/artesunate	21	63	52	11
Pyronaridine/artesunate	8	24	10	14
Chloroquine/amodiaquine	13	39	9	30
Azithromycin/pyronaridine	12	36	25	11
Total	136	408	234	174
Average	13.6	40.8	23.4	17.4

**TABLE 3:** Fractional curative doses

Fraction of single drug X dose	Fraction of single drug Y dose (n = 3 mice)	Combination FCD sum X+Y (n=3 mice)	Average of Combination FCD sum (n=3 mice)	Fraction of single drug Y dose (n = 6 mice)	Combination FCD sum X+Y (n=6 mice)	Average Combination FCD sum (n=6 mice)
Artesunate	Tafenoquine					
0.14	0.5	0.64	0.67	0.66	0.8	0.83
0.29	0.33	0.62		0.5	0.79	
0.57	0.17	0.74		0.33	0.9	
Artesunate	Amodiaquine					
0.14	0.8	0.94	0.86			0.89
0.29	0.4	0.69		0.6	0.89	
0.56	0.4	0.96				
Artesunate	Chloroquine					
0.29	0.58	0.87	0.81	0.66	0.95	0.9
0.42	0.33	0.75		0.42	0.84	
0.57	0.25	0.82		0.33	0.9	
Pyronaridine	Tafenoquine					
0.75	0.166	0.92	0.94	0.33	1.08	1.08
0.5	0.5	1.00				
0.25	0.666	0.92				
Pyronaridine	Amodiaquine					
0.5	0.05	0.55	0.72	0.1	0.6	0.93
0.25	0.4	0.65		0.6	0.85	
0.75	0.2	0.95		0.6	1.35	
Pyronaridine	Chloroquine					
0.75	0.166	0.92	0.92	0.25	1	1
0.75	0.25	1.00				
0.5	0.33	0.83				
Artesunate	Azithromycin					
0.8	0.14	0.94	0.95			1.04
0.6	0.43	1.03		0.8	1.23	
0.4	0.57	0.97		0.4	0.97	
0.2	0.57	0.77				
0.2	0.71	0.91		0.2	0.91	
0.2	0.86	1.06		0.2	1.06	
Artesunate	Pyronaridine					
0.14	0.75	0.89	1.00			1.04
0.29	0.75	1.04		0.75	1.04	
0.57	0.5	1.07				
Amodiaquine	Chloroquine					
0.166	0.8	0.97	1.07			1.13
0.33	0.8	1.13		0.33	1.13	
0.5	0.6	1.10				
Pyronaridine	Azithromycin					
0.2	0.75	0.95	1.05	0.6	1.35	1.17
0.6	0.5	1.10		0.6	1.1	
0.6	0.25	1.10		0.8	1.05	

Of the pyronaridine drug interactions tested, amodiaquine was the strongest in the traditional checkerboard pattern with a fractional curative dose of 0.93. The sum of the fractional curative dose is additive, like with tafenoquine and artesunate. Pyronaridine was also additive with tafenoquine, chloroquine, artesunate, and azithromycin in the 0.9–1.1 range. The combination of chloroquine and amodiaquine was predicted to be additive for fractional dose cure, and we observed a value of 1.13.

**FIG 4:**
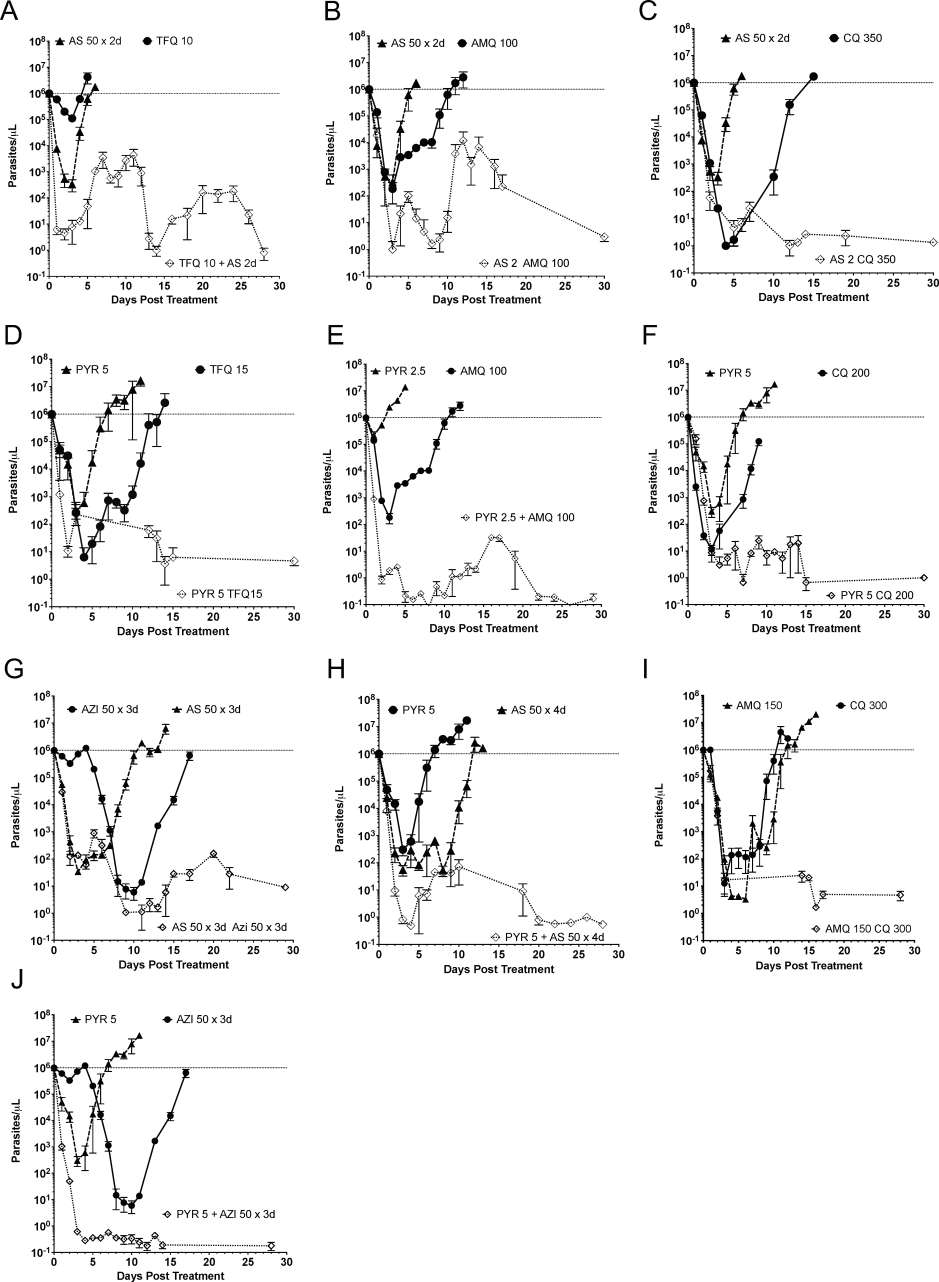
Early parasite clearance with time to return of initial parasitemia in single-drug subcurative doses compared to combination minimum curative doses. Pyronaridine/(tafenoquine, amodiaquine, or azithromycin) had an early more rapid combination drop in parasitemia. Combination pairs are tafenoquine/artesunate (**A**), amodiaquine/artesunate (**B**), chloroquine/artesunate (**C**), tafenoquine/pyronaridine (**D**), amodiaquine/pyronaridine (**E**), chloroquine/pyronaridine (**F**), azithromycin/artesunate (**G**), pyronaridine/artesunate (**H**), chloroquine/amodiaquine (**I**), and azithromycin/pyronaridine (**J**). Data points are represented as mean ± SEM (*n* = 3 mice).

**FIG 5:**
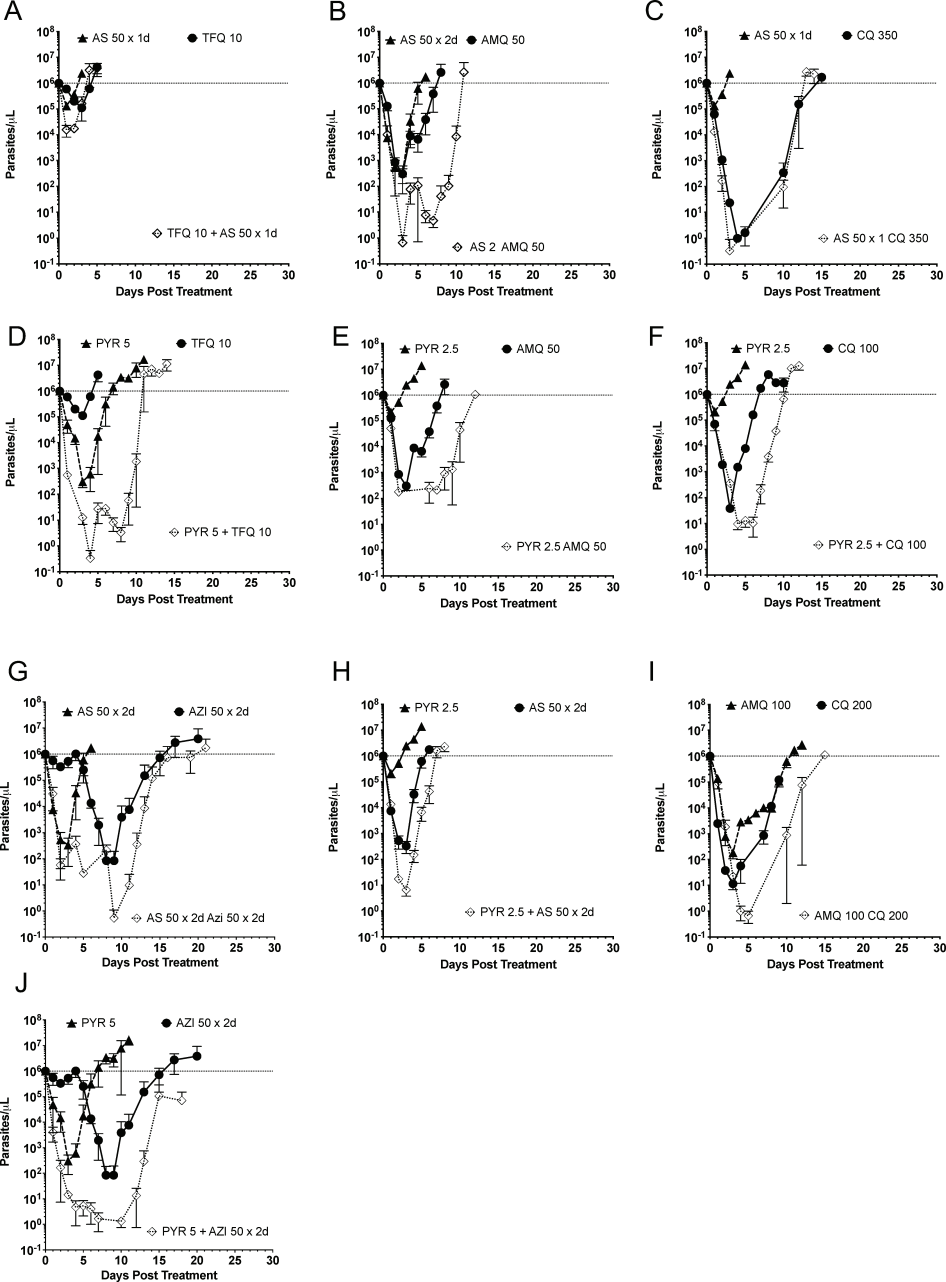
Early parasite clearance with time to return of initial parasitemia with noncurative combinations and single drugs. An early parasitemia drug combination drop in the case of pyronaridine/tafenoquine correlated to a delay in return to initial parasitemia, while just a delay in return without an early reduction was seen with pyronaridine with amodiaquine or chloroquine. Combination pairs are tafenoquine/artesunate (**A**), amodiaquine/artesunate (**B**), chloroquine/artesunate (**C**), tafenoquine/pyronaridine (**D**), amodiaquine/pyronaridine (**E**), chloroquine/pyronaridine (**F**), azithromycin/artesunate (**G**), pyronaridine/artesunate (**H**), chloroquine/amodiaquine (**I**), and azithromycin/pyronaridine (**J**). Data points are represented as mean ± SEM (*n* = 3 mice).

**FIG 6:**
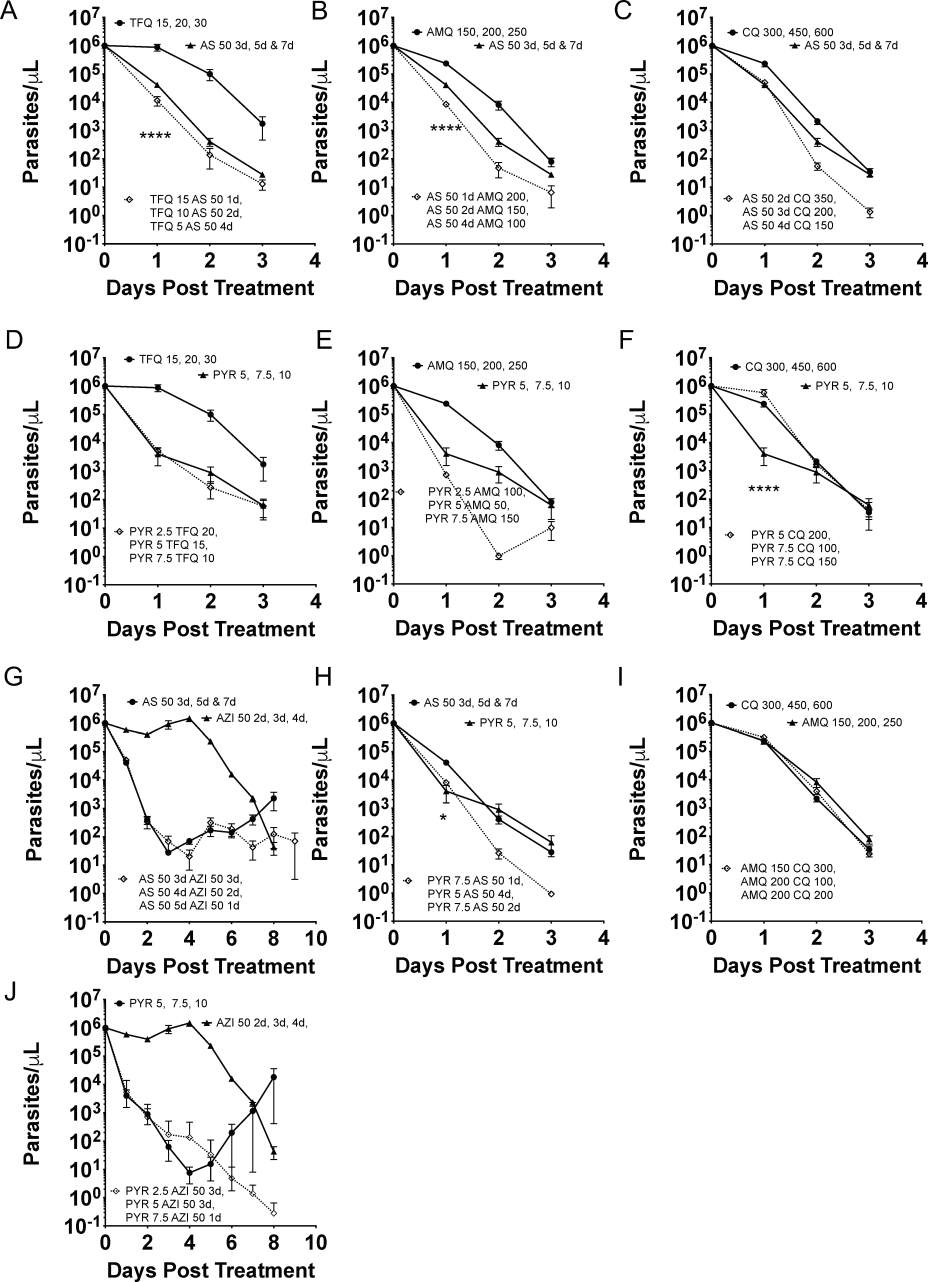
Parasite reduction with 50%, 75%, and 100% of single drugs and three minimum curative combinations. Artesunate with tafenoquine or amodiaquine showed a faster 24-hour reduction than either drug alone, while pyronaridine with amodiaquine or chloroquine was antagonistic in the 24-hour reduction. Combination pairs are tafenoquine/artesunate (**A**), amodiaquine/artesunate (**B**), chloroquine/artesunate (**C**), tafenoquine/pyronaridine (**D**), amodiaquine/pyronaridine (**E**), chloroquine/pyronaridine (**F**), azithromycin/artesunate (**G**), pyronaridine/artesunate (**H**), chloroquine/amodiaquine (**I**), and azithromycin/pyronaridine (**J**). Data points are represented as mean ± SEM (*n* = 9 mice)

The curative combination doses indicated a greater drop in parasitemia than single drugs among the first 5 days for tafenoquine/artesunate, amodiaquine/artesunate, pyronaridine/azithromycin, and pyronaridine/amodiaquine (Fig. 4). Except for the tafenoquine/pyronaridine combination and the azithromycin/pyronaridine combination, the noncurative combinations did not show a greater early parasitemia drop in the combination than either drug alone, which was also independently seen as a 1–2-day delay to initial parasitemia (Fig. 5). The pyronaridine/chloroquine or pyronaridine/amodiaquine combination did not show a change in early parasite clearance but manifested a 3-day delay in the combination parasitemia return. A more rigorous analysis combined three single drug doses at approximately 50%, 75%, and 100% of curative drug doses, as well as three curative combination doses, to characterize the parasite reduction ratios with nine mice in a group. This confirmed that early, day 1–3, greater fold parasitemia reductions were present for the more additive combination of tafenoquine/artesunate and pyronaridine/amodiaquine, as well as amodiaquine/artesunate (Fig. 6). Chloroquine was antagonistic with pyronaridine in initial parasite reduction. In this rough low number of drug combinations, the parasite reduction ratios were sensitive, but not specifically correlated to fractional curative dose (two of three). Early antagonism with chloroquine and pyronaridine was additive in curative combination. Azithromycin combination with either pyronaridine or artesunate was indifferent in the first three days. After 3 days, the parasitemia was low enough that the interactions were hard to interpret.

## DISCUSSION

This work provides a framework to evaluate pharmacodynamic interactions for malaria drugs *in vivo*. The most common bench metric is the *P. falciparum* inhibition assay *in vitro,* comparing untreated parasite fractions to drug-treated fractions with a low parasite inoculum (16). Modifications to measure true *P. falciparum* killing by dilution or unique reporters are more limited based on more involved complicated assays (17, 18). Most introductory murine malaria drug models also utilize a low-dose inoculum, comparing percent inhibition to untreated controls. Drugs can reduce parasite multiplication to one or two per time period lifecycle to show more than 90% inhibition. These do not always correlate to killing curative combination action in the high parasitemia case presentations.

Here, we compared combinations in a high parasitemia mouse model reliant on killing activity to reduce parasitemia. We measured parasite reduction ratios, followed by lags in return to initial million parasites per μL and curative activity. Cure rates are different from rapid reduction in parasitemia. Rapid reductions in parasitemia are necessary to save lives (19). Artesunate, despite ring-stage resistance, which leads to curative treatment failure, still saves lives with rapid reduction in parasitemia when compared to quinine (20). Our results were in the additive range of a fractional curative dose analysis.

Kevin Batty and colleagues also developed a high parasitemia pharmacodynamic model of initiating drug treatment near 2–5% followed by Giemsa blood films with an essentially 3 log window of quantification from 0.01% to 10% (21–23). The model was adapted to the *P. berghei* luciferase-expressing parasite with a 3–4 log range used to demonstrate long-duration artemisinins can make the difference between cure and no cure with the quinolines (24) and single-dose cures with the quinolines and the artemisinins, where pyronaridine had a more rapid killing than artesunate (25).

The majority of *Plasmodium* combination drug studies are reported as fractional inhibition concentrations in the traditional 48–72-hour continuous drug assays (26).

This work found only two combinations—tafenoquine/artesunate and pyronaridine/amodiaquine, which were more additive in fractional curative dosing. Tafenoquine was not approved in 2018 for use with the artemisinins (27). Tafenoquine is metabolized by the mouse even though active metabolites from cytochrome P450 2D targeting the malaria mitochondria might be different from human metabolites (28, 29). The parent drug has higher 50% inhibition concentrations *in vitro* against *P. falciparum* of approximately 0.1 µM (30). Tafenoquine and the artemisinins in combination *in vitro* assays previously indicated addition (31). A recent clinical trial showed antagonism of tafenoquine and primaquine with dihydroartemisinin-piperaquine for dormant human *P. vivax (9*).

How these *in vivo* studies will translate to human infections and treatment is important. Further studies will be required to test the findings from this study in humans, particularly how combination drug therapy upholds a synergistic and/or additive profile in malaria infection. Addressing the treatment response differences that exist between an *in vivo* murine model, humanized mouse models, and human infections is also important. Future research can address these pharmacodynamic differences with a call to implement paired quinolines with the artemisinins to reduce the threat of drug resistance.

## MATERIAL AND METHODS

### Drug preparation and dosing

The antimalarial drugs artesunate, pyronaridine tetraphosphate, chloroquine diphosphate, azithromycin hydrochloride, tafenoquine succinate, and amodiaquine dihydrochloride were obtained from Sigma-Aldrich. Artesunate is 100% parent drug. Artesunate was dissolved in 5% sodium bicarbonate. The others were dissolved in sterile water. Fresh drug solution was used for each experiment. For all the experimental studies done, each drug solution was dosed at a volume of 200 µL per mouse.

### *In vivo* cytocidal model of murine malaria

The rodent model used for all the experiments was Balb/cj female mice from the Jackson Laboratories aged at least 6 weeks old and weighed approximately 20 grams each. For each study done, replicates of three mice were used for each drug dose regimen tested. *P. berghei* ANKA, 676m1cl1, Green fluorescence protein-luciferase (PbANKA GFP-Luc) obtained from ATCC (catalog # MRA-868), constitutively expresses the luciferase at all stages in the life cycle. For each experiment, Balb/cj female mice were infected via intraperitoneal (i.p.) with approximately 100,000 erythrocytes infected with PbANKA GFP-Luc from a donor mouse between 5 to 10 % parasitemia (in first or second passage). For malaria blood-stage infections and drug responses, there are no sex differences in Balb/c mice. As male and female Balb/c mice display differences in tissue iron levels, female mice were used for consistency throughout all experiments (32).

### Luciferase assay and analysis

During the drug treatment, 5 µL of blood was collected from the tail of each mouse at regular intervals and deposited into 45 µL of lysis buffer in a 96-well plate (29). Samples were stored at −80°C until processed. A total of 5 µL of blood/lysis buffer (whole blood equivalent of 0.5 μL) was transferred to a black, opaque 96-well plate, and 95 µL of luciferase buffer (20 mM Tricine, 100 µM EDTA, 1.07 mM K_2_CO_3_, 2.67 mM MgSO_4_, 17 mM DTT, 250 µM ATP, 250 µM D-Luciferin) was added. Luciferase activity was measured in the IVIS Spectrum *In Vivo* Imaging System and analyzed using Living Image v. 4.4 software. The raw luciferase activity is expressed as radiant flux in photons/second. Total radiant flux was compared to parasites per well using GraphPad Prism 5 software.

### Data analysis

All the data analyses and representation were performed with GraphPad Prism 5 Software. Data are represented as mean ± SEM. The luciferase raw data log 10 total flux (photons/sec) was transformed to parasites per μL using the equation Y = (10^((log(y)−0.55)/0.05))*2 (24). All data were normalized to a mean parasitemia of 1 million parasites per μL at the time of drug treatment. The statistical methods employed include the following: Two-way analysis of variance (ANOVA) and/or *t*-test (non-parametric) were used for comparing two groups. For groups more than two, One-way ANOVA with Tukey Multiple comparison post-hoc test was used for comparison of groups as appropriate, with alpha significance level *P* < 0.05.

### Fractional curative dose

Minimum curative single drug doses for the quinolines and the allometric daily mouse dose in number of days for artesunate and azithromycin were determined. Tafenoquine was dosed at 5, 10, 15, and 20 mg/kg to represent 0.16, 0.33, 0.5, and 0.66 of the 30 mg/kg curative dose. Amodiaquine was dosed at 12.5, 25, 50, 100, and 200 mg/kg to represent 0.5, 0.1, 0.2, 0.4, 0.6, and 0.8 of the 250 mg/kg curative dose. Chloroquine was dosed at 50, 100, 150, 200, 250, 300, 350, 400, and 450 mg/kg for corresponding fractions of the 600 mg/kg curative dose. Pyronaridine was dosed at 1.25, 2.5, 5, and 7.5 mg/kg for corresponding 0.125, 0.25, 0.5, and 0.75 of the 10 mg/kg curative dose. Artesunate at 50 mg/kg was dosed by number of days −1, 2, 3, 4, 5, or 6 to represent multiples of 0.14 of the 7-day curative dose. Azithromycin also at 50 mg/kg was dose for 1, 2, 3, or 4 days to represent 0.2 fractions of the curative dose of 5 days. The fractional curative dose adds the fraction of single drug-X curative dose used in combination with the other fraction of single drug-Y curative dose for each point along the curve (33). The fractions are added for each drug combination for the combination sum. The multiple points are averaged for the fractional curative dose (33). Synergy would be a fractional curative dose less than 0.5. Additive is in the range of 0.51–1.99 with antagonism over 2.0 (34).
